# Ethyl 2-[(*E*)-({2,4-dimeth­oxy-6-[2-(4-meth­oxy­phen­yl)ethen­yl]benzyl­idene}amino)­oxy]acetate

**DOI:** 10.1107/S2414314621009500

**Published:** 2021-09-17

**Authors:** Jiha Sung

**Affiliations:** a Department of Applied Chemistry, Dongduk Women’s University, Seoul 136-714, Republic of Korea; University of Aberdeen, Scotland

**Keywords:** crystal structure, resveratrol, oxime ester, C—H⋯O hydrogen bonds

## Abstract

In the title compound, C_22_H_25_NO_6_, the C=C double bond linking the benzene rings adopts an *E* configuration and the dihedral angle between the rings is 47.1 (2)°. The oxime unit contains a C=N double bond, which also has an *E* configuration. In the crystal, pairs of C—H⋯N hydrogen bonds generate inversion dimers and weak C—H⋯O inter­actions link the dimers into chains propagating along the *b*-axis direction.

## Abstract 2

The crystal structure of a resveratrol–oxime ester is reported.

## Graphical abstract

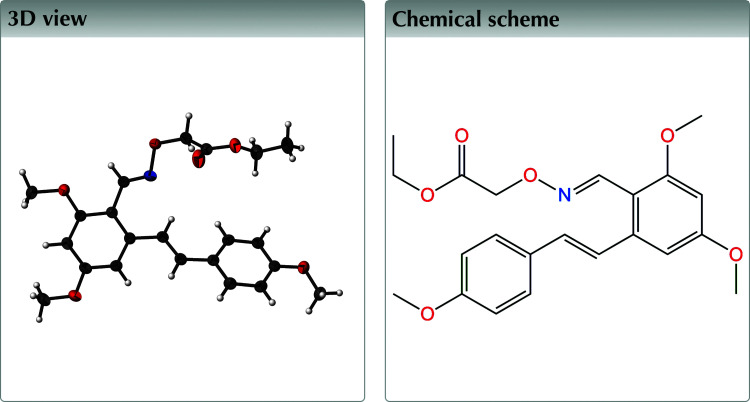

## Structure description

A recent review has demonstrated that chemically modified resveratrol derivatives have diverse biological activities (Li *et al.*, 2019 ▸). Oxime esters are one of the most important pharmacophores in a large number of bioactive compounds (Vessally *et al.*, 2016 ▸). As part of our studies in this area, *O*-methyl­ated resveralol aldehyde (Ge *et al.*, 2013 ▸) was treated with hydroxyl­amine to give the corresponding oxime analogue, which was reacted with ethyl bromo­acetate to provide the title resveratrol-oxime ester compound.

**Figure 1:**
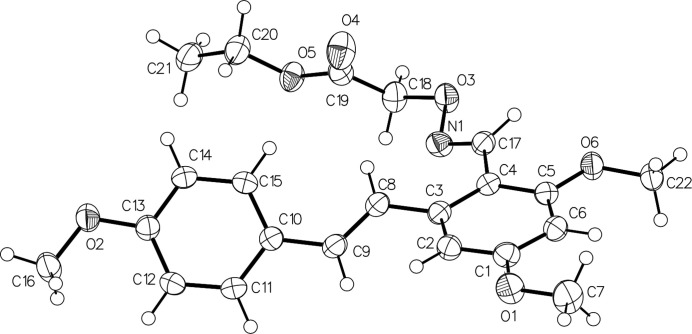
The mol­ecular structure of the title compound with displacement ellipsoids drawn at the 30% probability level.

**Figure 2:**
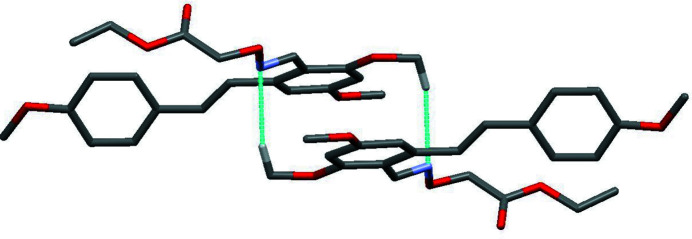
A view of the inversion dimer formed by a pair of C—H⋯N hydrogen bonds (dashed lines) in the crystal structure of the title compound, generating an 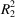 (14) loop. For clarity, only those H atoms involved in hydrogen bonding are shown.

**Figure 3:**
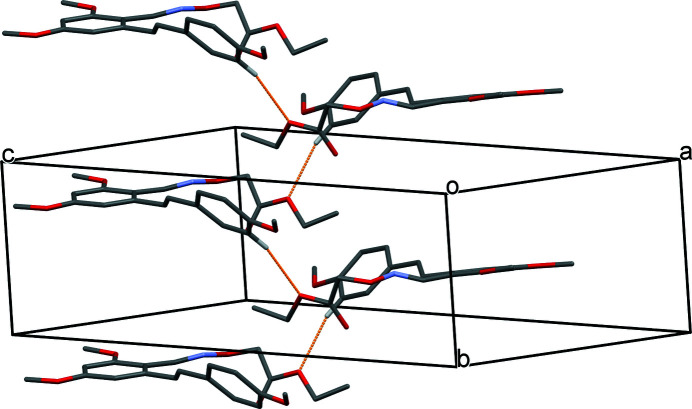
Part of the crystal structure showing C—H⋯O hydrogen bonds as orange dashed lines. For clarity, only those H atoms involved in hydrogen bonding are shown.

The mol­ecular structure of the title compound, C_22_H_25_NO_6_, is shown in Fig. 1 ▸. The benzene rings (C1–C6 and C10–C15) are connected by the C8=C9 double bond, which has an *E*-configuration [torsion angle of 173.69 (12)° for C3—C8—C9—C10]. The dihedral angle formed by benzene rings is 47.1 (2)°. The C17=N1 imine double bond in the oxime unit also adopts an *E* configuration, which is defined by a torsion angle of 178.3 (1)° for C4—C17—N1—O3. There are three meth­oxy groups attached to carbon atoms C1, C5 and C13 in the benzene rings: those at the *meta* positions (C1, C5) are essentially co-planar with their attached benzene rings [C6—C1—O1—C7 = −0.2 (2)° and C6—C5—O6—C22 = 3.9 (2)°] whereas the meth­oxy group at the *para* position (C13) is slightly twisted from the corresponding ring plane [C12—C13—O2—C16 = 8.9 (2)°]. In the crystal, pairs of C22—H22⋯N1 hydrogen bonds generate inversion dimers (Table 1 ▸, Fig. 2 ▸) and the C14—H14⋯O5 hydrogen bond links the dimers into chains propagating along the *b*-axis direction (Table 1 ▸, Fig. 3 ▸).

## Synthesis and crystallization

A mixture of *E*-2,4-dimeth­oxy-6-(4-meth­oxy­styr­yl)benz­aldehyde (298 mg, 1 mmol; Ge *et al.*, 2013 ▸) and hydroxyl­amine hydro­chloride (69 mg, 1 mmol) in 15 ml of ethanol–water (1:1) was refluxed for 4 h. After completion of reaction, the mixture was cooled to room temperature to give the corresponding oxime derivative (86%, m.p. = 150–152°C), which was used for the next reaction. To a mixture of the oxime derivative (156 mg, 0.5 mmol) and potassium carbonate (276 mg, 2 mmol) in 10 ml of DMF, 1.2 equivalents of ethyl bromo­acetate (100 mg, 0.6 mmol) were added and heated for 5 h at 60°C. After completion of the reaction, the reaction mixture was poured into crushed ice–water to form a precipitate. The resulting solid was separated by filtration and was washed with ethyl acetate. Recrystallization of the solid from ethyl acetate solution gave colourless blocks of the title compound.

## Refinement

Crystal data, data collection and structure refinement details are summarized in Table 2 ▸.

## Supplementary Material

Crystal structure: contains datablock(s) I. DOI: 10.1107/S2414314621009500/hb4391sup1.cif

Structure factors: contains datablock(s) I. DOI: 10.1107/S2414314621009500/hb4391Isup2.hkl

Supporting information file. DOI: 10.1107/S2414314621009500/hb4391Isup3.cml

CCDC reference: 2109339

Additional supporting information:  crystallographic information; 3D view; checkCIF report

## Figures and Tables

**Table 1:** Hydrogen-bond geometry (Å, °)

*D*—H⋯*A*	*D*—H	H⋯*A*	*D*⋯*A*	*D*—H⋯*A*
C22—H22*C*⋯N1^i^	0.98	2.61	3.5633 (19)	166
C14—H14⋯O5^ii^	0.95	2.55	3.4834 (16)	166

Symmetry codes: (i) 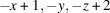 ; (ii) 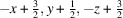 .

**Table 2:** Experimental details

Crystal data	
Chemical formula	C_22_H_25_NO_6_
*M*_r_	399.43
Crystal system, space group	Monoclinic, *P*2_1_/*n*
Temperature (K)	193
*a*, *b*, *c* (Å)	11.3656 (9), 7.0636 (5), 26.035 (2)
β (°)	100.148 (3)
*V* (Å^3^)	2057.4 (3)
*Z*	4
Radiation type	Mo *K*α
μ (mm^−1^)	0.09
Crystal size (mm)	0.36 × 0.19 × 0.10

Data collection	
Diffractometer	PHOTON 100 CMOS
No. of measured, independent and observed [*I* > 2σ(*I*)] reflections	72604, 5157, 4367
*R*_int_	0.052
(sin θ/λ)_max_ (Å^−1^)	0.670

Refinement	
*R*[*F* ^2^ > 2σ(*F* ^2^)], *wR*(*F* ^2^), *S*	0.046, 0.133, 1.06
No. of reflections	5157
No. of parameters	266
H-atom treatment	H-atom parameters constrained
Δρ_max_, Δρ_min_ (e Å^−3^)	0.32, −0.19

Computer programs: *APEX2* and *SAINT* (Bruker, 2012 ▸), *SHELXS* and *SHELXTL* (Sheldrick, 2008 ▸), *SHELXL2014* (Sheldrick, 2015 ▸) and *publCIF* (Westrip, 2010 ▸).

## full crystallographic data

### Crystal data

**Table:**

C_22_H_25_NO_6_	*F*(000) = 848
*M**_r_* = 399.43	*D*_x_ = 1.290 Mg m^−^^3^
Monoclinic, *P*2_1_/*n*	Mo *K*α radiation, λ = 0.71073 Å
Hall symbol: -P 2yn	Cell parameters from 9916 reflections
*a* = 11.3656 (9) Å	θ = 2.4–28.2°
*b* = 7.0636 (5) Å	µ = 0.09 mm^−^^1^
*c* = 26.035 (2) Å	*T* = 193 K
β = 100.148 (3)°	Block, colourless
*V* = 2057.4 (3) Å^3^	0.36 × 0.19 × 0.10 mm
*Z* = 4	

### Data collection

**Table:**

PHOTON 100 CMOS diffractometer	4367 reflections with *I* > 2σ(*I*)
Radiation source: fine-focus sealed tube	*R*_int_ = 0.052
Graphite monochromator	θ_max_ = 28.4°, θ_min_ = 2.1°
φ and ω scans	*h* = −15→15
72604 measured reflections	*k* = −9→9
5157 independent reflections	*l* = −34→34

### Refinement

**Table:**

Refinement on *F*^2^	Primary atom site location: structure-invariant direct methods
Least-squares matrix: full	Secondary atom site location: difference Fourier map
*R*[*F*^2^ > 2σ(*F*^2^)] = 0.046	Hydrogen site location: inferred from neighbouring sites
*wR*(*F*^2^) = 0.133	H-atom parameters constrained
*S* = 1.06	*w* = 1/[σ^2^(*F*_o_^2^) + (0.0616*P*)^2^ + 0.781*P*] where *P* = (*F*_o_^2^ + 2*F*_c_^2^)/3
5157 reflections	(Δ/σ)_max_ = 0.001
266 parameters	Δρ_max_ = 0.32 e Å^−^^3^
0 restraints	Δρ_min_ = −0.18 e Å^−^^3^

### Special details

**Table:**

Geometry. All esds (except the esd in the dihedral angle between two l.s. planes) are estimated using the full covariance matrix. The cell esds are taken into account individually in the estimation of esds in distances, angles and torsion angles; correlations between esds in cell parameters are only used when they are defined by crystal symmetry. An approximate (isotropic) treatment of cell esds is used for estimating esds involving l.s. planes.
Refinement. Refinement of F^2^ against ALL reflections. The weighted R-factor wR and goodness of fit S are based on F^2^, conventional R-factors R are based on F, with F set to zero for negative F^2^. The threshold expression of F^2^ > 2sigma(F^2^) is used only for calculating R-factors(gt) etc. and is not relevant to the choice of reflections for refinement. R-factors based on F^2^ are statistically about twice as large as those based on F, and R- factors based on ALL data will be even larger.

### Fractional atomic coordinates and isotropic or equivalent isotropic displacement parameters (Å^2^)

**Table:**

	*x*	*y*	*z*	*U*_iso_*/*U*_eq_	
C1	0.24155 (11)	0.2768 (2)	0.96981 (5)	0.0339 (3)	
C2	0.27638 (11)	0.2914 (2)	0.92163 (5)	0.0330 (3)	
H2	0.2175	0.3046	0.8911	0.040*	
C3	0.39681 (11)	0.28708 (17)	0.91753 (5)	0.0289 (2)	
C4	0.48435 (11)	0.26856 (17)	0.96321 (5)	0.0277 (2)	
C5	0.44554 (11)	0.25016 (17)	1.01146 (5)	0.0292 (3)	
C6	0.32511 (12)	0.25393 (19)	1.01513 (5)	0.0331 (3)	
H6	0.3005	0.2411	1.0480	0.040*	
O1	0.12111 (9)	0.28900 (18)	0.96906 (4)	0.0448 (3)	
C7	0.08045 (14)	0.2768 (3)	1.01766 (7)	0.0531 (4)	
H7A	0.1001	0.1517	1.0331	0.080*	
H7B	−0.0063	0.2954	1.0120	0.080*	
H7C	0.1196	0.3748	1.0413	0.080*	
C8	0.42894 (11)	0.31089 (18)	0.86556 (5)	0.0300 (3)	
H8	0.5016	0.3741	0.8633	0.036*	
C9	0.36217 (12)	0.24905 (18)	0.82140 (5)	0.0318 (3)	
H9	0.2942	0.1749	0.8247	0.038*	
C10	0.38336 (11)	0.28418 (17)	0.76843 (5)	0.0286 (2)	
C11	0.31500 (12)	0.18938 (19)	0.72667 (5)	0.0345 (3)	
H11	0.2584	0.0980	0.7335	0.041*	
C12	0.32681 (12)	0.2241 (2)	0.67544 (5)	0.0349 (3)	
H12	0.2800	0.1555	0.6478	0.042*	
C13	0.40742 (11)	0.35951 (19)	0.66487 (5)	0.0316 (3)	
C14	0.47781 (12)	0.4548 (2)	0.70595 (5)	0.0353 (3)	
H14	0.5341	0.5465	0.6989	0.042*	
C15	0.46637 (11)	0.41688 (19)	0.75671 (5)	0.0326 (3)	
H15	0.5158	0.4820	0.7843	0.039*	
O2	0.42356 (9)	0.40901 (17)	0.61580 (4)	0.0428 (3)	
C16	0.34007 (14)	0.3337 (2)	0.57350 (5)	0.0425 (3)	
H16A	0.3487	0.1958	0.5726	0.064*	
H16B	0.3554	0.3877	0.5406	0.064*	
H16C	0.2587	0.3658	0.5781	0.064*	
C17	0.61356 (11)	0.27478 (18)	0.96420 (5)	0.0303 (3)	
H17	0.6643	0.3104	0.9957	0.036*	
N1	0.66105 (10)	0.23457 (17)	0.92473 (5)	0.0345 (3)	
O3	0.78758 (8)	0.25945 (16)	0.93779 (4)	0.0390 (2)	
C18	0.83580 (12)	0.2301 (2)	0.89191 (5)	0.0375 (3)	
H18A	0.8035	0.1106	0.8752	0.045*	
H18B	0.9236	0.2164	0.9014	0.045*	
C19	0.80732 (11)	0.3908 (2)	0.85321 (5)	0.0339 (3)	
O4	0.75832 (12)	0.53568 (17)	0.86054 (4)	0.0531 (3)	
O5	0.84641 (9)	0.34797 (14)	0.80913 (4)	0.0372 (2)	
C20	0.83492 (14)	0.4957 (2)	0.76952 (6)	0.0411 (3)	
H20A	0.8764	0.6119	0.7842	0.049*	
H20B	0.7496	0.5263	0.7574	0.049*	
C21	0.88974 (16)	0.4244 (2)	0.72495 (6)	0.0469 (4)	
H21A	0.9724	0.3852	0.7379	0.070*	
H21B	0.8890	0.5253	0.6991	0.070*	
H21C	0.8437	0.3159	0.7087	0.070*	
O6	0.53333 (8)	0.22915 (15)	1.05405 (4)	0.0364 (2)	
C22	0.49803 (13)	0.1985 (2)	1.10347 (5)	0.0378 (3)	
H22A	0.4497	0.3057	1.1116	0.057*	
H22B	0.5693	0.1868	1.1306	0.057*	
H22C	0.4508	0.0819	1.1021	0.057*	

### Atomic displacement parameters (Å^2^)

**Table:**

	*U*^11^	*U*^22^	*U*^33^	*U*^12^	*U*^13^	*U*^23^
C1	0.0266 (6)	0.0382 (7)	0.0379 (7)	−0.0006 (5)	0.0081 (5)	−0.0013 (5)
C2	0.0281 (6)	0.0381 (7)	0.0321 (6)	−0.0002 (5)	0.0032 (5)	0.0024 (5)
C3	0.0298 (6)	0.0278 (6)	0.0294 (6)	−0.0010 (4)	0.0064 (5)	0.0012 (4)
C4	0.0272 (6)	0.0263 (5)	0.0301 (6)	−0.0011 (4)	0.0064 (5)	0.0013 (4)
C5	0.0289 (6)	0.0291 (6)	0.0292 (6)	−0.0016 (5)	0.0043 (5)	0.0006 (4)
C6	0.0322 (6)	0.0374 (7)	0.0311 (6)	−0.0022 (5)	0.0094 (5)	−0.0001 (5)
O1	0.0264 (5)	0.0684 (7)	0.0408 (6)	0.0001 (5)	0.0090 (4)	−0.0012 (5)
C7	0.0330 (7)	0.0821 (13)	0.0477 (9)	−0.0010 (7)	0.0162 (6)	−0.0021 (8)
C8	0.0271 (6)	0.0304 (6)	0.0326 (6)	0.0004 (5)	0.0061 (5)	0.0048 (5)
C9	0.0303 (6)	0.0316 (6)	0.0341 (6)	−0.0029 (5)	0.0074 (5)	0.0039 (5)
C10	0.0268 (6)	0.0281 (6)	0.0309 (6)	0.0015 (4)	0.0045 (5)	0.0018 (5)
C11	0.0333 (6)	0.0345 (6)	0.0358 (7)	−0.0100 (5)	0.0061 (5)	−0.0003 (5)
C12	0.0334 (6)	0.0379 (7)	0.0321 (6)	−0.0081 (5)	0.0022 (5)	−0.0044 (5)
C13	0.0273 (6)	0.0373 (7)	0.0304 (6)	−0.0002 (5)	0.0057 (5)	0.0016 (5)
C14	0.0312 (6)	0.0397 (7)	0.0350 (6)	−0.0109 (5)	0.0054 (5)	0.0017 (5)
C15	0.0297 (6)	0.0348 (6)	0.0322 (6)	−0.0067 (5)	0.0019 (5)	−0.0017 (5)
O2	0.0401 (5)	0.0590 (7)	0.0291 (5)	−0.0104 (5)	0.0056 (4)	0.0025 (4)
C16	0.0421 (8)	0.0555 (9)	0.0295 (6)	−0.0012 (7)	0.0051 (5)	−0.0033 (6)
C17	0.0284 (6)	0.0322 (6)	0.0300 (6)	−0.0013 (5)	0.0047 (5)	0.0033 (5)
N1	0.0249 (5)	0.0428 (6)	0.0359 (6)	−0.0018 (4)	0.0051 (4)	−0.0024 (5)
O3	0.0242 (4)	0.0627 (7)	0.0298 (5)	−0.0004 (4)	0.0043 (4)	0.0036 (4)
C18	0.0289 (6)	0.0509 (8)	0.0338 (7)	0.0070 (6)	0.0088 (5)	0.0046 (6)
C19	0.0295 (6)	0.0409 (7)	0.0321 (6)	0.0007 (5)	0.0077 (5)	−0.0026 (5)
O4	0.0706 (8)	0.0481 (6)	0.0457 (6)	0.0193 (6)	0.0240 (6)	0.0018 (5)
O5	0.0441 (5)	0.0364 (5)	0.0340 (5)	0.0062 (4)	0.0152 (4)	0.0023 (4)
C20	0.0522 (8)	0.0357 (7)	0.0373 (7)	0.0025 (6)	0.0135 (6)	0.0047 (6)
C21	0.0599 (10)	0.0449 (8)	0.0402 (7)	−0.0093 (7)	0.0206 (7)	0.0001 (6)
O6	0.0310 (5)	0.0507 (6)	0.0272 (4)	−0.0017 (4)	0.0044 (4)	0.0043 (4)
C22	0.0400 (7)	0.0465 (8)	0.0268 (6)	−0.0043 (6)	0.0055 (5)	−0.0001 (5)

### Geometric parameters (Å, º)

**Table:**

C1—O1	1.3683 (16)	C14—C15	1.3768 (18)
C1—C2	1.3842 (18)	C14—H14	0.9500
C1—C6	1.3876 (19)	C15—H15	0.9500
C2—C3	1.3919 (17)	O2—C16	1.4242 (17)
C2—H2	0.9500	C16—H16A	0.9800
C3—C4	1.4158 (17)	C16—H16B	0.9800
C3—C8	1.4719 (17)	C16—H16C	0.9800
C4—C5	1.4089 (17)	C17—N1	1.2742 (17)
C4—C17	1.4649 (17)	C17—H17	0.9500
C5—O6	1.3628 (15)	N1—O3	1.4291 (14)
C5—C6	1.3890 (18)	O3—C18	1.4144 (16)
C6—H6	0.9500	C18—C19	1.515 (2)
O1—C7	1.4242 (18)	C18—H18A	0.9900
C7—H7A	0.9800	C18—H18B	0.9900
C7—H7B	0.9800	C19—O4	1.1962 (17)
C7—H7C	0.9800	C19—O5	1.3362 (15)
C8—C9	1.3345 (18)	O5—C20	1.4565 (17)
C8—H8	0.9500	C20—C21	1.498 (2)
C9—C10	1.4623 (17)	C20—H20A	0.9900
C9—H9	0.9500	C20—H20B	0.9900
C10—C11	1.3917 (18)	C21—H21A	0.9800
C10—C15	1.4012 (17)	C21—H21B	0.9800
C11—C12	1.3857 (18)	C21—H21C	0.9800
C11—H11	0.9500	O6—C22	1.4302 (15)
C12—C13	1.3853 (18)	C22—H22A	0.9800
C12—H12	0.9500	C22—H22B	0.9800
C13—O2	1.3676 (15)	C22—H22C	0.9800
C13—C14	1.3913 (18)		
			
O1—C1—C2	115.30 (12)	C13—C14—H14	119.8
O1—C1—C6	123.55 (12)	C14—C15—C10	121.36 (12)
C2—C1—C6	121.14 (12)	C14—C15—H15	119.3
C1—C2—C3	120.65 (12)	C10—C15—H15	119.3
C1—C2—H2	119.7	C13—O2—C16	116.50 (11)
C3—C2—H2	119.7	O2—C16—H16A	109.5
C2—C3—C4	119.52 (11)	O2—C16—H16B	109.5
C2—C3—C8	118.33 (11)	H16A—C16—H16B	109.5
C4—C3—C8	122.09 (11)	O2—C16—H16C	109.5
C5—C4—C3	118.25 (11)	H16A—C16—H16C	109.5
C5—C4—C17	117.23 (11)	H16B—C16—H16C	109.5
C3—C4—C17	124.47 (11)	N1—C17—C4	123.02 (12)
O6—C5—C6	122.35 (11)	N1—C17—H17	118.5
O6—C5—C4	115.86 (11)	C4—C17—H17	118.5
C6—C5—C4	121.79 (12)	C17—N1—O3	109.39 (11)
C1—C6—C5	118.61 (12)	C18—O3—N1	107.72 (10)
C1—C6—H6	120.7	O3—C18—C19	112.50 (11)
C5—C6—H6	120.7	O3—C18—H18A	109.1
C1—O1—C7	117.64 (12)	C19—C18—H18A	109.1
O1—C7—H7A	109.5	O3—C18—H18B	109.1
O1—C7—H7B	109.5	C19—C18—H18B	109.1
H7A—C7—H7B	109.5	H18A—C18—H18B	107.8
O1—C7—H7C	109.5	O4—C19—O5	124.44 (13)
H7A—C7—H7C	109.5	O4—C19—C18	125.86 (12)
H7B—C7—H7C	109.5	O5—C19—C18	109.70 (11)
C9—C8—C3	123.98 (12)	C19—O5—C20	116.35 (11)
C9—C8—H8	118.0	O5—C20—C21	108.06 (12)
C3—C8—H8	118.0	O5—C20—H20A	110.1
C8—C9—C10	126.42 (12)	C21—C20—H20A	110.1
C8—C9—H9	116.8	O5—C20—H20B	110.1
C10—C9—H9	116.8	C21—C20—H20B	110.1
C11—C10—C15	117.15 (12)	H20A—C20—H20B	108.4
C11—C10—C9	119.55 (11)	C20—C21—H21A	109.5
C15—C10—C9	123.24 (11)	C20—C21—H21B	109.5
C12—C11—C10	122.11 (12)	H21A—C21—H21B	109.5
C12—C11—H11	118.9	C20—C21—H21C	109.5
C10—C11—H11	118.9	H21A—C21—H21C	109.5
C13—C12—C11	119.53 (12)	H21B—C21—H21C	109.5
C13—C12—H12	120.2	C5—O6—C22	117.86 (10)
C11—C12—H12	120.2	O6—C22—H22A	109.5
O2—C13—C12	124.35 (12)	O6—C22—H22B	109.5
O2—C13—C14	116.15 (11)	H22A—C22—H22B	109.5
C12—C13—C14	119.49 (12)	O6—C22—H22C	109.5
C15—C14—C13	120.32 (12)	H22A—C22—H22C	109.5
C15—C14—H14	119.8	H22B—C22—H22C	109.5
			
O1—C1—C2—C3	177.99 (12)	C9—C10—C11—C12	176.62 (13)
C6—C1—C2—C3	−1.2 (2)	C10—C11—C12—C13	−1.2 (2)
C1—C2—C3—C4	−0.4 (2)	C11—C12—C13—O2	−177.91 (13)
C1—C2—C3—C8	−177.58 (12)	C11—C12—C13—C14	1.9 (2)
C2—C3—C4—C5	1.62 (18)	O2—C13—C14—C15	178.88 (12)
C8—C3—C4—C5	178.74 (11)	C12—C13—C14—C15	−0.9 (2)
C2—C3—C4—C17	−175.79 (12)	C13—C14—C15—C10	−0.8 (2)
C8—C3—C4—C17	1.32 (19)	C11—C10—C15—C14	1.4 (2)
C3—C4—C5—O6	178.79 (11)	C9—C10—C15—C14	−175.53 (13)
C17—C4—C5—O6	−3.61 (17)	C12—C13—O2—C16	8.9 (2)
C3—C4—C5—C6	−1.41 (18)	C14—C13—O2—C16	−170.87 (13)
C17—C4—C5—C6	176.20 (12)	C5—C4—C17—N1	158.35 (13)
O1—C1—C6—C5	−177.70 (13)	C3—C4—C17—N1	−24.2 (2)
C2—C1—C6—C5	1.4 (2)	C4—C17—N1—O3	178.31 (11)
O6—C5—C6—C1	179.69 (12)	C17—N1—O3—C18	−174.86 (11)
C4—C5—C6—C1	−0.1 (2)	N1—O3—C18—C19	72.08 (14)
C2—C1—O1—C7	−179.36 (14)	O3—C18—C19—O4	5.7 (2)
C6—C1—O1—C7	−0.2 (2)	O3—C18—C19—O5	−174.87 (11)
C2—C3—C8—C9	−33.37 (19)	O4—C19—O5—C20	4.5 (2)
C4—C3—C8—C9	149.49 (13)	C18—C19—O5—C20	−174.88 (12)
C3—C8—C9—C10	173.69 (12)	C19—O5—C20—C21	176.76 (12)
C8—C9—C10—C11	171.46 (13)	C6—C5—O6—C22	3.90 (18)
C8—C9—C10—C15	−11.6 (2)	C4—C5—O6—C22	−176.29 (11)
C15—C10—C11—C12	−0.5 (2)		

### Hydrogen-bond geometry (Å, º)

**Table:**

*D*—H···*A*	*D*—H	H···*A*	*D*···*A*	*D*—H···*A*
C22—H22*C*···N1^i^	0.98	2.61	3.5633 (19)	166
C14—H14···O5^ii^	0.95	2.55	3.4834 (16)	166

Symmetry codes: (i) −*x*+1, −*y*, −*z*+2; (ii) −*x*+3/2, *y*+1/2, −*z*+3/2.
